# Rhein ameliorates transverse aortic constriction-induced cardiac hypertrophy *via* regulating STAT3 and p38 MAPK signaling pathways

**DOI:** 10.3389/fphar.2022.940574

**Published:** 2022-08-26

**Authors:** Run-Jing Li, Jia-Jia Xu, Zheng-Hao Zhang, Min-Wei Chen, Shi-Xiao Liu, Cui Yang, Yan-Ling Li, Ping Luo, Yi-Jiang Liu, Rong Tang, Zhong-Gui Shan

**Affiliations:** ^1^ Department of Cardiology, Xiamen Key Laboratory of Cardiac Electrophysiology, Xiamen Institute of Cardiovascular Diseases, The First Affiliated Hospital of Xiamen University, School of Medicine, Xiamen University, Xiamen, China; ^2^ Department of Cardiac Surgery, Xiamen Key Laboratory of Cardiac Electrophysiology, Xiamen Institute of Cardiovascular Diseases, The First Affiliated Hospital of Xiamen University, School of Medicine, Xiamen University, Xiamen, China

**Keywords:** rhein, transverse aortic constriction, cardiac hypertrophy, cardiac fibrosis, ERK phosphorylation, p38 MAPK, STAT

## Abstract

The progression from compensatory hypertrophy to heart failure is difficult to reverse, in part due to extracellular matrix fibrosis and continuous activation of abnormal signaling pathways. Although the anthraquinone rhein has been examined for its many biological properties, it is not clear whether it has therapeutic value in the treatment of cardiac hypertrophy and heart failure. In this study, we report for the first time that rhein can ameliorate transverse aortic constriction (TAC)-induced cardiac hypertrophy and other cardiac damage *in vivo* and *in vitro*. In addition, rhein can reduce cardiac hypertrophy by attenuating atrial natriuretic peptide, brain natriuretic peptide, and β-MHC expression; cardiac fibrosis; and ERK phosphorylation and transport into the nucleus. Furthermore, the inhibitory effect of rhein on myocardial hypertrophy was similar to that of specific inhibitors of STAT3 and ERK signaling. In addition, rhein at therapeutic doses had no significant adverse effects or toxicity on liver and kidney function. We conclude that rhein reduces TAC-induced cardiac hypertrophy via targeted inhibition of the molecular function of ERK and downregulates STAT3 and p38 MAPK signaling. Therefore, rhein might be a novel and effective agent for treating cardiac hypertrophy and other cardiovascular diseases.

## Introduction

Cardiac hypertrophy is an early feature and a key factor in heart failure and sudden death (Messerli et al., 2017). Cardiac hypertrophy is accompanied by increases in protein synthesis, numbers of sarcomeres, cardiomyocyte diameter or length, and production of fibrous tissue. During myocardial hypertrophy, myocardial oxygen consumption increases, fetal genes are re-expressed, electrophysiological abnormalities appear, and myocardial cells are remodeled (Molkentin et al., 1998; Burchfield et al., 2013). In addition, diastolic and systolic dysfunction occurs, and fibroblasts, vascular smooth muscle cells, and endothelial cells also undergo complex stress responses that lead to inflammatory cell infiltration, endothelial dysfunction, and excessive accumulation of extracellular matrix, all of which promote fibrosis. In myocardial cells, hypertrophy is associated with apoptosis (Prabhu et al., 2003) and disrupted electrical conduction that leads to arrhythmias. Transverse aortic constriction (TAC) in mice is a commonly used experimental model for pressure overload-induced cardiac hypertrophy and heart failure. Chronic hemodynamic overload leads to cardiac decompensation (deAlmeida et al., 2010). Recent studies have shown that MAPK signaling can be activated by hemodynamic changes acting on the ventricular wall (Aikawa et al., 2002; Brancaccio et al., 2006) as a result of TAC-induced cardiac hypertrophy. However, specific MAPK/ERK inhibitors can attenuate this process (Vallentin and Mochly-Rosen, 2007). Moreover, mechanical stretching can upregulate phosphorylation of STAT3 (Ruppert and Meyer, 2007). Many experiments have shown that STAT3 and p38 MAPK are central to the development of cardiac hypertrophy. In addition, angiotensin II (AngII) can activate the AT1 receptor both *in vivo* and *in vitro* to mediate cardiac hypertrophy (Hernandez-Hernandez et al., 2002; Lee et al., 2020). Although a large number of studies have described the pathophysiological mechanism of the progression of cardiac hypertrophy to heart failure in mammals, a lack of effective treatment for cardiac hypertrophy still remains. Therefore, finding new therapeutic approaches for the treatment of cardiac hypertrophy is critically important for reversing one of the most frequent causes of heart failure.

Rhein (4-5-dihydroxyanthraquinone-2-carboxylic acid) is one of the main active components extracted from rhubarb and other traditional Chinese herbs such as *Polygonum multiflorum*. It is a monoanthracene derivative of 1,8-dihydroxyanthraquinone (Barbosa et al., 2020). Rhein has a wide range of pharmacological activities against tumors (Lin et al., 2009; Cho et al., 2017; Zhang et al., 2020), inflammation (Ge et al., 2017; Wu et al., 2020), fibrosis (Zhang et al., 2016), oxidative stress (Zhao et al., 2018), and renal disease (Hu et al., 2020), and can be a part of treatments for diabetic nephropathy (Wang et al., 2019), ischemia-reperfusion injury (Liu et al., 2018), influenza (Wang et al., 2018), and many other diseases. Wang et al. (2020) showed that rhein promoted apoptosis of HepG2 and Huh7 cells by regulating ROS/JNK/caspase-3 signaling pathway, thus inhibiting the occurrence and development of liver cancer. In addition, other experiments have shown that rhein can reverse cell apoptosis induced by hydrogen peroxide and inhibit the production of ROS, thus protecting cells from oxidative stress. This study also proved that rhein could achieve this function by regulating the PI3K/AKT signaling pathway (Zhuang et al., 2019a). It is worth noting that many reports have confirmed that rhein can inhibit tumor cell proliferation and protect the functioning of many organs by inhibiting inflammation and fibrosis and downregulating activation of the STAT3 and MAPK signaling pathways (Ma et al., 2018; Zhuang et al., 2019b; Yang et al., 2019). However, the therapeutic effect of rhein on cardiac hypertrophy and its possible mechanisms remain unclear. Therefore, this study used AngII and TAC to induce cardiac hypertrophy *in vitro* and *in vivo*, respectively, to study the inhibitory effect of rhein on cardiac hypertrophy and its molecular mechanisms.

## Materials and methods

### Animals and TAC operation

Male C57BL/6 mice of 20–25 g were purchased from SLAC Biological Company (Shanghai, China). The mice housed in an SPF environment and were fed at a constant temperature and humidity. TAC operation was performed in a clean surgery room, and the operating field was disinfected with 75% alcohol. Mice were anesthetized in an induction chamber with 2% isoflurane mixed with 0.5–1.0 L/min 100% O_2_. The fur was shaved from the neckline to mid-chest level. Thoracotomy to the second rib under a surgical microscope was performed and the sternum retracted using a chest retractor. Meanwhile, following the identification of the transverse aorta, a 6.0 silk suture overlying a 27G needle was placed between the innominate and left carotid arteries. Then two knots were tied quickly, and the needle promptly removed to yield a constriction of 0.4 mm diameter. In sham control mice, the entire procedure was the same except for the ligation of the aorta. Rhein was dissolved in normal saline, and the mice were given 200 μl gradient gavage of rhein once a day. The experiment was conducted randomly on six groups with six mice in each group, namely, the control group, TAC group, 50 mg/kg rhein group, 150 mg/kg rhein group, 200 mg/kg rhein group, and positive control group of 10 mg/kg telmisartan (cat. no. A8531, APExBIO Technology, Houston, Texas, United States). Tissue sampling was studied 28 days later.

### Echocardiography measurement

Mice were anesthetized with 2% isoflurane mixed with 0.5–1.0 L/min 100% O_2_, the anterior chest hair was shaved, and an ultrasonic coupling agent was applied evenly to the model 550 probe and chest. IVSd, LVIDd, LVPWd, IVSs, LVIDs, LVPWs, EF, and FS were measured by VisualSonics high-resolution Vevo 2100 system (VisualSonics, Toronto, Canada).

### Cell culture

H9C2 rat cardiomyocytes were purchased from Procell Life Science & Technology Company (Wuhan, China). AC16 human cardiomyocytes were purchased from BLUEFBIO Science Company (Shanghai, China). All cell lines were cultured in high-glucose DMEM, supplemented with 10% fetal bovine serum (FBS), penicillin, and streptomycin. All cell lines were maintained in a 5% CO_2_ humidified cell incubator at 37 °C. The cells were treated with 1 μM angiotensin II (Xu et al., 2021).

### MTT assay

MTT assay was utilized to measure H9C2 and AC16 cell cytotoxicity and viability. H9C2 and AC16 cells were dispensed in 96-well plates at 5x10^3^ cells per well and allowed to attach overnight, then the cells were treated with rhein (APExBIO cat. no. N1810, United States ; purity = 99.75%) at test concentrations (0, 1, 10, 50, 125, 250, 500, and 1000 μM) for 24 h; and then, subsequently, 20 μL MTT solution was added at a concentration of 5 mg/ml and the mixture incubated at 37°C for 4 h. Then, 10% SDS 100 μL was added and mixed for 12 h to dissolve the formed formazan crystals, and the optical density (OD) of each well was measured at 490 nm using a microplate reader. Cell viability was calculated using the following formula: viability = (average OD values of treatment wells/average OD values of vehicle control wells) × 100%.

### Histopathology

The isolated heart was fixed with 4% paraformaldehyde for 24 h. The dehydrated tissue was embedded in paraffin and cut into 5 μm thick sections. Hematoxylin and eosin (H&E) staining was used to evaluate myocardial structural changes. Masson tricolor staining kit (cat. no. D026-1-3, Nanjing, China) was used to evaluate the degree of fibrosis. The micrographs were enlarged to ×400 using an intelligent biological microscope (OLYMPUS.BX53). A quantitative digital image analysis system (Image-Pro Plus 6.0 software) was used to calculate the percentage of collagen staining (blue).

### Wheat germ agglutinin (WGA)

The sections were stained with 50 μg/ml WGA for 60 min and observed with a fluorescence inverted microscope at ×400 magnification. The number of cells in a fixed area per slide was measured, and the average cell size was calculated using Image-J analysis software.

### Immunofluorescence

All rhein-treated cell lines were fixed with 4% paraformaldehyde for 30 min then permeated membranes with 0.1% Triton X-100. Subsequently, H9C2 and AC16 cells were incubated with P-ERK antibody (cat. no. 4370T, Cell Signaling Technology) at 4 °C overnight and then with the fluorescent secondary antibody Alexa Fluor 488 (cat. no. A0423, Beyotime Institute of Biotechnology, Shanghai, China) for 1 h at 37 °C. The nucleus was stained with DAPI (cat. no. S2110, Solarbio Science & Technology Co., Ltd, Beijing, China). F-actin was stained with actin-tracker red-rhodamine (cat. no. C2207S, Beyotime Institute of Biotechnology, Shanghai, China).

### Nucleo-plasmic separation

About 5 × 10^6^ H9C2 cells were washed with PBS and resuspended in 200 l of buffer A (10 mM HEPES (pH 7.9), 10 mM KCl, 1.5 mM MgCl_2_, 0.34 M sucrose, 10% glycerol, 1 mM dithiothreitol, 0.1% Triton X-100, and protease inhibitor mixture (Roche Molecular Biochemicals)). The cells were incubated for 15 min on ice. Then the supernatant containing cytoplasm was collected at 13,000 ×g, 10 min, 4°C. Protein concentrations were measured by the BCA method. Nuclei were washed once with buffer A without 0.1% Triton X-100 and then lysed in 50 l of buffer B (3 mM EDTA, 0.2 mM EGTA, 1 mM dithiothreitol, and protease inhibitor mixture). After the 15-min incubation on ice, soluble nuclear proteins were separated from chromatin by centrifugation (2000 ×g, 4 min). Then chromatin was washed once with buffer B, and the supernatant was collected at high speed (13,000 ×g, 1 min). The protein concentration was determined by the BCA method, and the sample was boiled and loaded, sample buffer: 6 × 8 ml sample (0.5M Tri 1 ml, glycerol 1.6 ml, 10% SDS 1.6 ml, 0.5% bromophenol 0.8 ml, β-ME 0.4 ml, and H_2_O 2.6 ml).

### Total RNA extraction and real-time PCR

Total RNA was isolated by using Trizol reagent (Takara, Japan), transcribed into cDNA with Takara reverse transcription kit Prime Script RT Master Mix (cat. no. RR036A). According to the manufactures’ instructions, mRNA levels of genes were quantitatively examined by RT-PCR using Fast SYBR™ Green Master Mix (cat. no. 4385610, Thermo Fisher, Waltham, MA, United States ) in a fluorescence quantitative PCR instrument (BIO RAD CFX96). The relative expression of genes was quantified by the 2^−ΔΔCt^ method. GAPDH was taken as an internal reference. For the primer sequence, refer to the supplementary data (Supplementary Table S1).

### Western blot

The mice left ventricle tissue, H9C2 cells, and AC16 cells were homogenized in RIPA lysis buffer (Beyotime Institute of Biotechnology, Shanghai, China). The concentrations of protein were determined using the BCA protein kit (Beyotime, China). Western blotting was performed following a standard protocol, and the blots were incubated with primary antibodies such as GAPDH (cat. no. 60004-1-Ig, Proteintech), ANP (cat. no. ab180649, Abcam), BNP (cat. no. ab239510, Abcam), COL3A1 (cat. no. 30565S, Cell Signaling Technology), COL1A1 (cat. no. 72026T, Cell Signaling Technology), β-MHC (cat. no. ab-50967, Abcam), STAT3 (cat. no. 9139S, Cell Signaling Technology), p-STAT3 (cat. no. 9145S, Cell Signaling Technology), p38 (cat. no. 8690, Cell Signaling Technology), p-p38 (cat. no. 4511, Cell Signaling Technology), JNK (cat. no. 9252, Cell Signaling Technology), p-JNK (cat. no. 4668, Cell Signaling Technology), ERK1/2 (cat. no. 137F5, Cell Signaling Technology), and P-ERK1/2 (cat. no. 4370, Cell Signaling Technology), at 4 °C overnight. After washing with TBST thrice, the membranes were incubated with horseradish peroxidase-conjugated goat anti-rabbit or goat anti-mouse secondary antibodies at room temperature for 1 h. Results were detected using the ECL detection kit (Millipore, United States).

### Serum biochemical indexes

Blood was collected from mice eyeballs, centrifuged at 4000 RPM for 10 min at 4 °C overnight, and the supernatant was separated. The following biochemical indexes were examined from experimental mice serum samples: liver function indexes [alanine aminotransferase (ALT), aspartate aminotransferase (AST), kidney function indexes [urea formaldehyde (UREA), creatinine sox (CREA-S)], and heart function indexes [lactate dehydrogenase (LDH)]. All the indexes were detected by an automatic chemistry analyzer (BS-240vet, Mindray Bio-Medical Electronics Co. Ltd., Shenzhen, China).

### Statistical analysis

All results are presented as the mean ± SD and analyzed using GraphPad (version 8.0.1, GraphPad Prism software). Unpaired *t*-test and one-way analysis of variance (ANOVA) by Tukey^’^s multiple comparisons test were used to compare each variable for differences among the groups. The statistical significance was expressed as *p* < 0.05.

## Results

### Rhein attenuates cardiomyocyte hypertrophy induced by AngII *in vitro*

**FIGURE 1:**
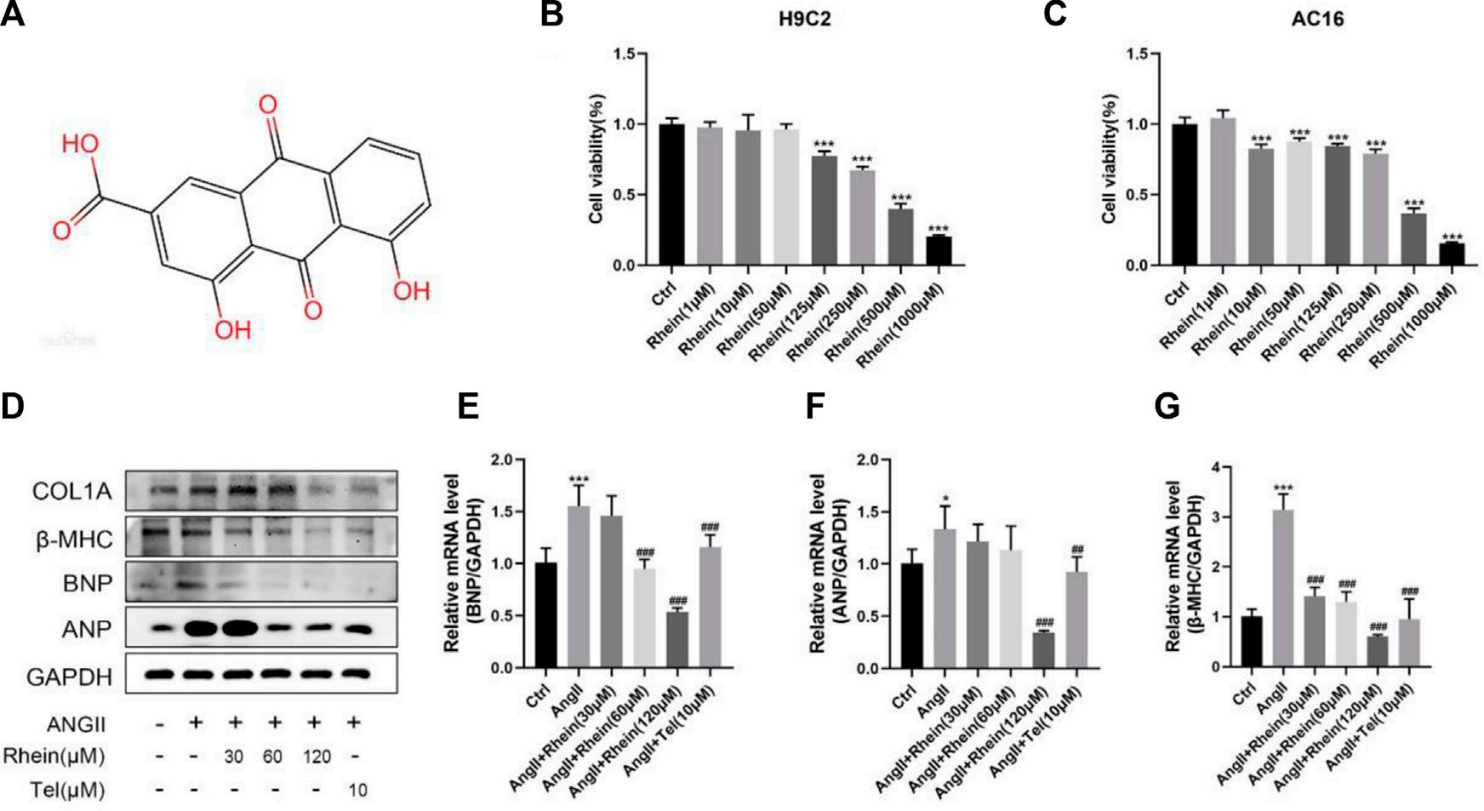
Rhein attenuates cardiomyocyte hypertrophy induced by AngII *in vitro.* **(A)** Chemical structure of rhein. **(B,C)** H9C2 and AC16 cells were stimulated with rhein (0–1,000 μM) for 24 h. Cell viabilities were measured by the MTT assay. **(D)** Relative expression of ANP, BNP, and β-MHC proteins in H9C2 cells treated with AngII, rhein, and telmisartan. **(E–G)** Quantitative PCR (qPCR) showing the mRNA levels of AngII-induced hypertrophic markers, ANP, BNP, and β-MHC, using RT-PCR analysis. Data are mean ± SD (n ≥ 3). *p < 0.05, **p < 0.01, ***p < 0.001 vs Ctrl group, #*p* < 0.05, ##*p* < 0.01, ###*p* < 0.001 vs AngII group (analyzed using one-way ANOVA). Telmisartan (Tel) is the positive control group.

Rhein is a monomer substance, and its chemical structure is shown in Figure 1A. The MTT assay was used to study rhein toxicity and its effects on the proliferation of H9C2 and AC16 cells; compared with the control group (0 µM rhein), toxicity was low in cells exposed to a low concentration of rhein. Based on these results, 30, 60, and 120 μM rhein were selected for subsequent experiments (Figures 1B, C).

AngII is an important component of the renin–angiotensin system (RAS), and it can strongly constrict blood vessels, leading to cardiac hypertrophy by causing an increase in cardiac afterload. H9C2 cells were incubated with 1 μM AngII to stimulate cardiomyocyte hypertrophy. Since the effect of rhein on hypertrophic H9C2 and AC16 cells has not been studied, we investigated whether rhein had a protective effect on myocardial hypertrophy at the cellular level. First, we examined a marker of cardiomyocyte hypertrophy, atrial natriuretic peptide (ANP), and analyzed BNP (brain natriuretic peptide) and β-MHC (β-myosin heavy chain) levels in control and rhein-treated cells in response to AngII treatment using RT-PCR and Western blot analysis. When AngII was used to stimulate H9C2 and AC16 cells, expression of ANP, BNP, and β-MHC mRNA were significantly upregulated. Exposure of these cells to rhein significantly reversed this effect in a concentration-dependent manner (Figures 1E–G; Supplementary Figure S1) and also significantly reduced the expression of ANP, BNP, and β-MHC proteins (Figure 1D; Supplementary Figure S1A). Overall, our results demonstrate that rhein alleviates AngII-induced cardiomyocyte hypertrophy *in vitro*.

### Rhein can reduce hypertrophy by inhibiting phosphorylation and nuclear localization of ERK

**FIGURE 2:**
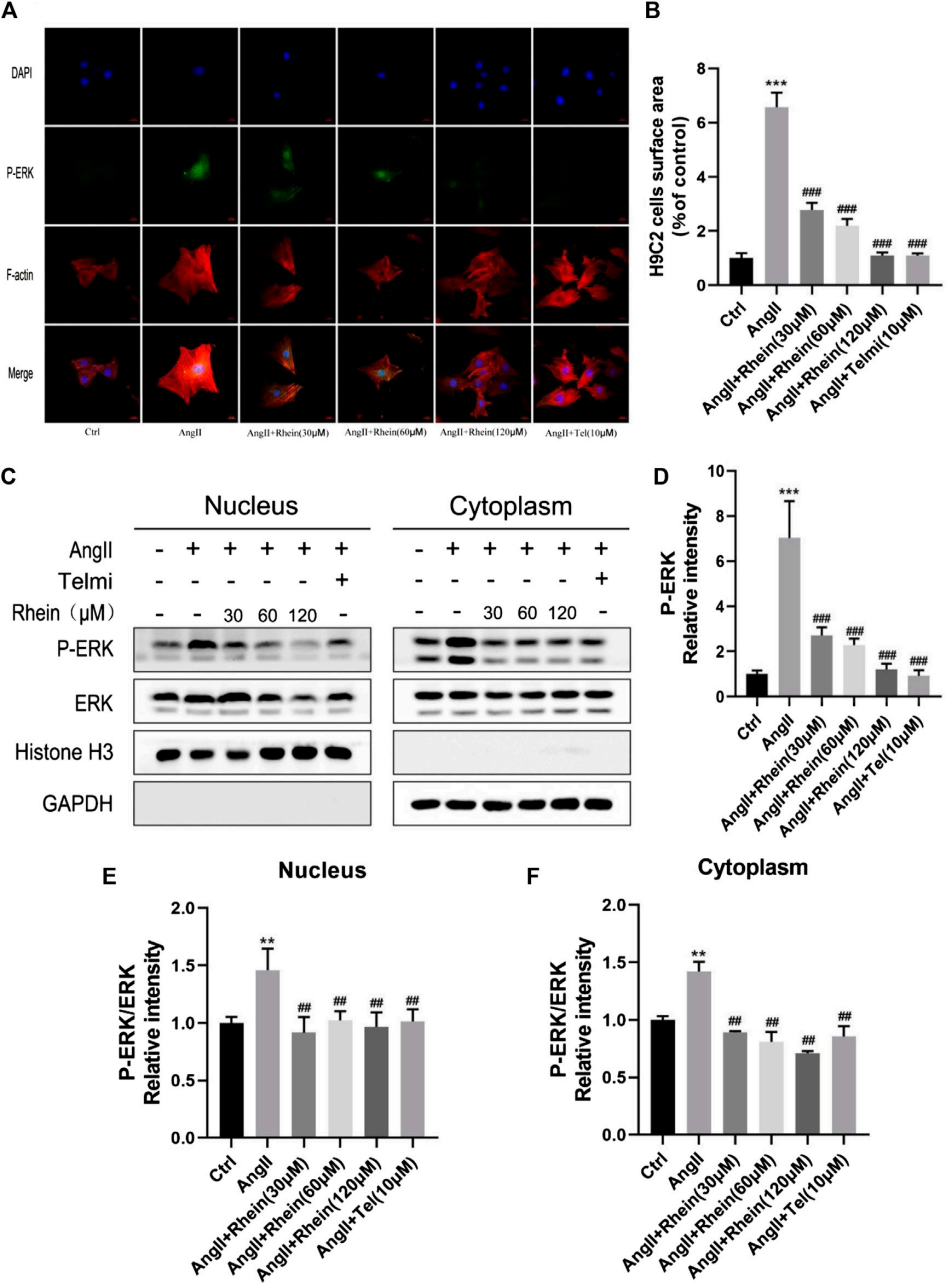
Rhein inhibits phosphorylation and nucleation of ERK to alleviate hypertrophy. **(A)** H9C2 cells stained with F-actin (red), DAPI (blue), and P-ERK (green) to detect the size of cells and relative intensity of P-ERK, and immunofluorescence images were captured by fluorescence microscopy. Scale bar = 50 µm. **(B)** Statistical quantitative analysis of the area of H9C2 cells by Image-Pro Plus software. **(C)** Nucleo-cytoplasmic separation assay was used to detect the effect of rhein on AngII-stimulated P-ERK nuclear translocation in H9C2 cells. **(D)** Statistical quantitative analysis of the relative fluorescence intensity of P-ERK in H9C2 cells by Image-Pro Plus software. **(E,F)** Relative strength of P-ERK in the nucleus and cytoplasm, respectively. Data are mean ± SD (n ≥ 3). *p < 0.05, **p < 0.01, ***p < 0.001 vs Ctrl group, #*p* < 0.05, ##*p* < 0.01, ###*p* < 0.001 vs AngII group (analyzed using one-way ANOVA).

One of the most important causes of myocardial hypertrophy is hemodynamic pressure overload acting on the walls of the ventricle. Activation of G protein-coupled receptors by neurohumoral factors or mechanical stress can activate MAPK family signaling pathways, which are involved with ERK and the emergency activating proteins SAPK/JNK and P38 kinase. In addition, AngII, as a key effector peptide of vasoconstriction and activation of the RAS, plays an important role in inducing hypertension and myocardial hypertrophy. Therefore, H9C2 cells were treated with ANGII (1 μM) and rhein (0, 30, 60, and 120 μM) for 24 h. First, we assessed cardiomyocyte size and ERK phosphorylation downstream of the MAPK pathway induced by rhein and telmisartan with or without AngII by immunofluorescence microscopy. Under the fluorescence microscope, F-actin appeared bright red, DAPI blue, and phosphorylated ERK (P-ERK) green (Figure 2A). The size (projected area) of H9C2 cells and relative intensity of P-ERK staining were quantified using Image-Pro Plus software (Figures 2B, D). Cell size increased significantly following AngII treatment, while the rhein-treated group exhibited a dose-dependent decrease. The relative density of P-ERK exhibited a similar decrease (seen with telmisartan treatment as a positive control). In conclusion, rhein can effectively inhibit AngII-induced cardiomyocyte hypertrophy and ERK phosphorylation *in vitro*.

Next, we determined whether rhein treatment affected nuclear translocation of ERK in H9C2 cells. Stimulation of H9C2 cells with 1 μM AngII resulted in the expected increase in nuclear levels of P-ERK, as shown by stronger nuclear green fluorescence than in unstimulated cells. As shown in Figure 2C, when H9C2 cells were stimulated by rhein, nuclear translocation of P-ERK was inhibited and cytoplasm levels of P-ERK were reduced (Figures 2E, F). These results further showed that rhein could inhibit AngII-induced ERK phosphorylation and translocation into the nucleus in myocardial cell lines.

### Rhein inhibits AngII-induced activation of STAT3 and p38/MAPK signaling pathways *in vitro*

**FIGURE 3:**
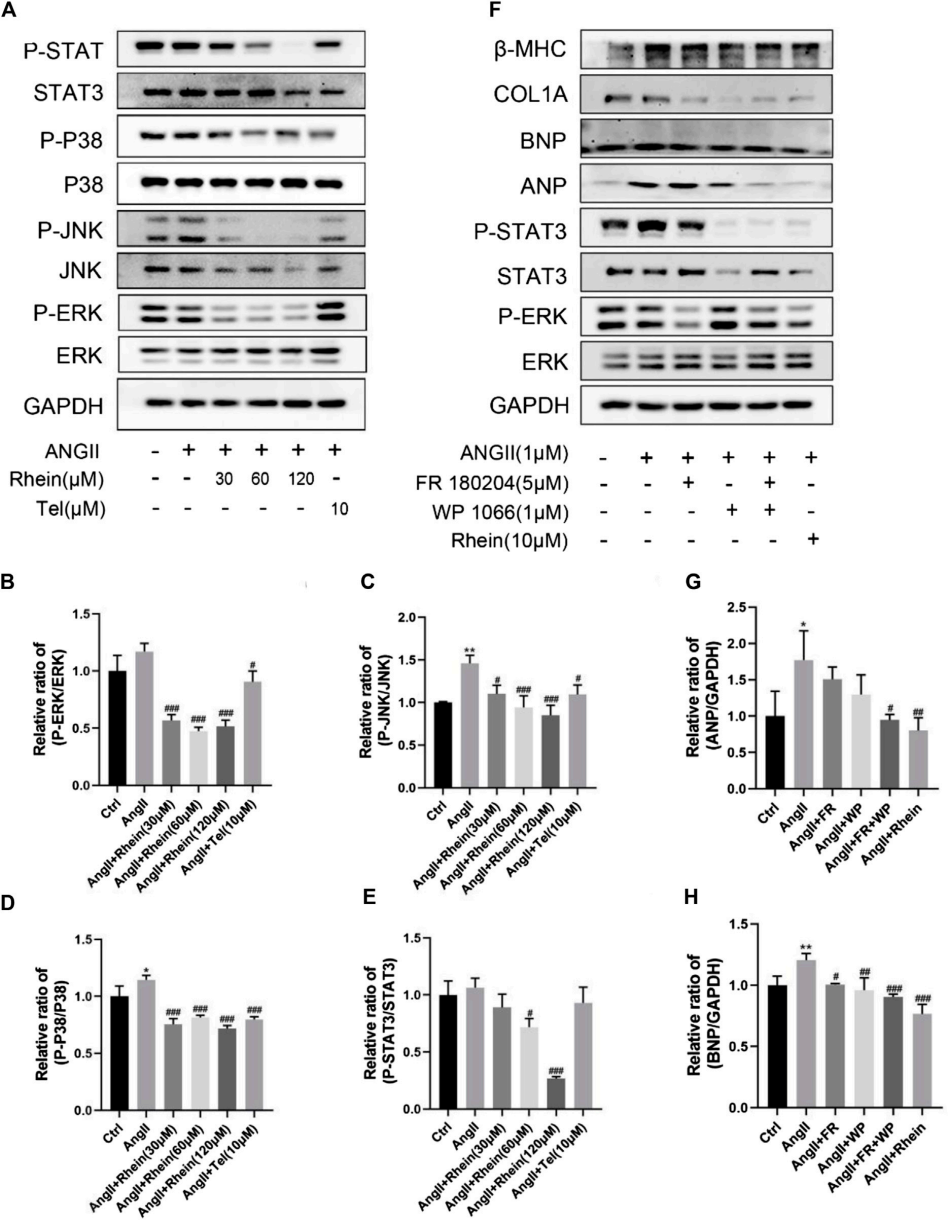
Rhein targets inhibition of AngII-stimulated activation of STAT3 and p38/MAPK signaling pathways *in vitro*. **(A)** Expression levels of signaling pathway proteins (total and phosphorylation of STAT3, p38, JNK, and ERK) were determined by Western blot in H9C2 cells. **(B–E)** Quantification of the relative changes in phosphorylation of STAT3, p38, JNK, and ERK. **(F)** The inhibitory effect of rhein on AngII-induced cardiac hypertrophy was compared with that of p-STAT3- and P-ERK-specific inhibitors. **(G,H)** Quantification of the relative changes in protein expression of ANP and BNP treated with specific inhibitors and rhein in cardiomyocytes. GAPDH was used for normalization. Data are mean ± SD (n ≥ 3). *p < 0.05, **p < 0.01, ***p < 0.001 vs Ctrl group, #*p* < 0.05, ##*p* < 0.01, ###*p* < 0.001 vs AngII group (analyzed using one-way ANOVA).

To investigate the mechanism of the effects of rhein on cardiac hypertrophy, we examined its effects on STAT3 and P38/MAPK signaling in AngII-induced hypertrophy and proliferation of H9C2 and AC16 cells. We found that AngII treatment resulted in significantly increased phosphorylation of STAT3, P38, JNK, and ERK proteins, which were significantly reduced after rhein treatment (Figure 3A; Supplementary Figure S2A). Meanwhile, statistical analysis showed that the P-STAT3/STAT3, P-P38/P38, P-JNK/JNK, and P-ERK/ERK ratios were significantly reduced after rhein treatment over a range of concentrations (Figures 3B–E; Supplementary Figures S2B–E). These results suggested that rhein inhibits AngII-induced activation of STAT3 and P38/MAPK signaling by inhibiting phosphorylation of key proteins. Subsequently, we further investigated whether rhein targets specific steps in the STAT3 and P38/MAPK signaling pathways. To our knowledge, specific inhibitors of STAT3 and ERK phosphorylation can antagonize AngII-induced cardiac hypertrophy. Therefore, we used WP1066 (Beyotime, China), a specific inhibitor of STAT3 phosphorylation, and FR180204 (Beyotime, China), a specific inhibitor of ERK phosphorylation, to provide evidence that rhein inhibits the myocardial hypertrophy cell phenotype by targeting p-STAT3 and P-ERK (Figure 3F). Interestingly, we found that rhein suppressed the cardiac hypertrophy phenotype more than the two inhibitors. Therefore, we suggest that rhein inhibits AngII-induced cardiac hypertrophy by targeting both the STAT3 and the P38/MAPK signaling pathways.

### Rhein reduces cardiac hypertrophy and cardiac fibrosis induced by TAC in wild-type mice

**FIGURE 4:**
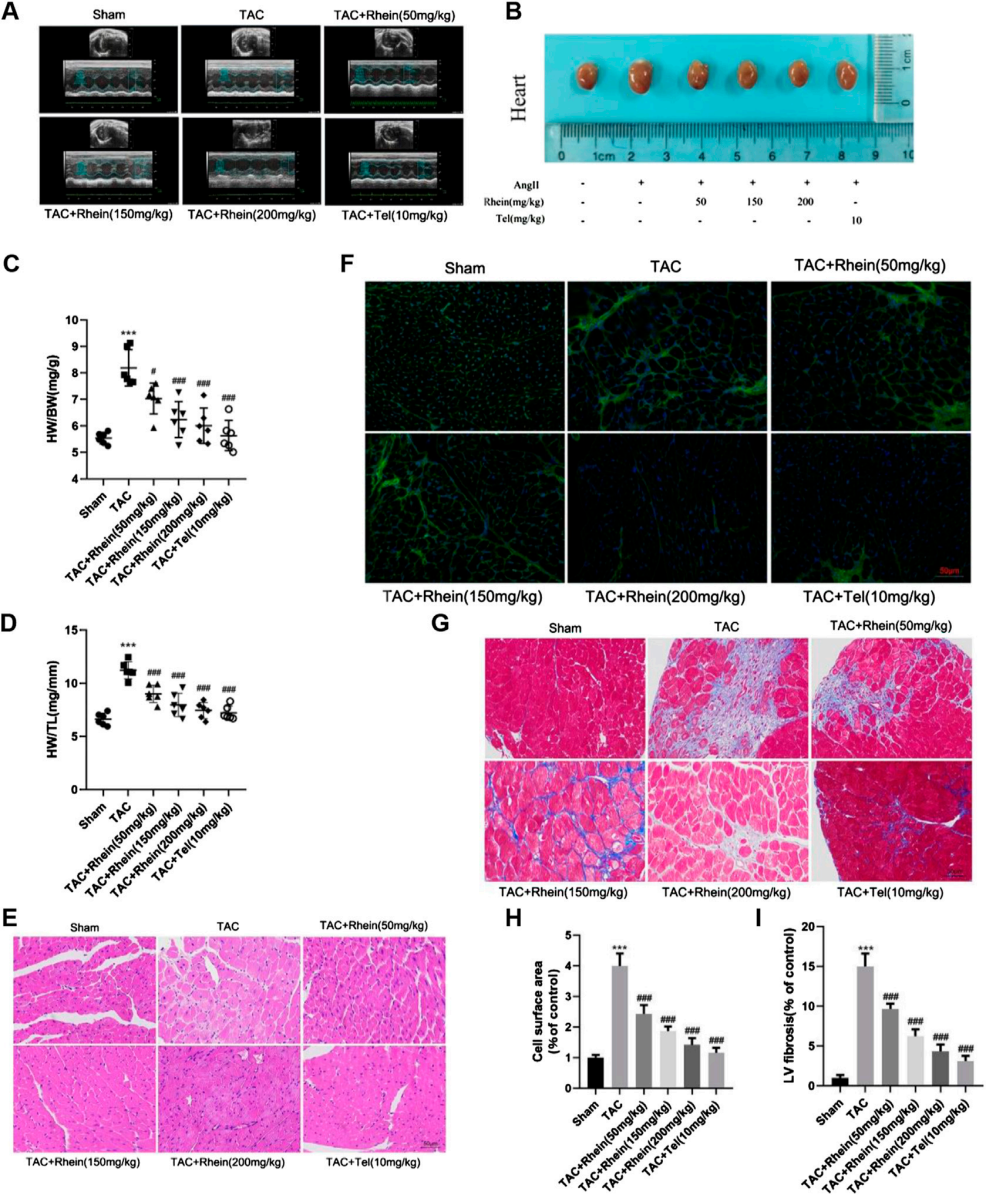
Rhein alleviates cardiac hypertrophy and cardiac fibrosis induced by TAC in WT mice. **(A)** Representative peak hemodynamics pictures. **(B)** Representative echocardiography pictures. **(C)** Morphological images of mouse hearts were exhibited from the sham, TAC, TAC + rhein (50 mg/kg), TAC + rhein (150 mg/kg), TAC + rhein (200 mg/kg), and TAC + Tel (10 mg/kg) groups. **(D)** Ratio of heart weight (HW) to body weight (BW). **(E)** Ratio of HW to tibia length in each group. **(F)** Paraffin sections of cardiac tissues were stained with H&E. Representative images are shown. Scale bar: 50 μm. **(G)** Myocardial cytoskeleton shows green fluorescence by WGA staining. Scale bar: 50 μm. **(H)** Masson’s trichrome staining of the hypertrophic heart. Collagen fibers are stained blue, and the myocardium is stained red. Scale bar: 50 μm. **(I)** Cell surface areas were quantified in the hearts of mice using Image-Pro Plus software. **(J)**Left ventricle fibrosis areas (of control) were quantified by Image-Pro Plus software. Data are mean ± SD (n = 6). *p < 0.05, **p < 0.01, ***p < 0.001 vs sham group, #*p* < 0.05, ##*p* < 0.01, ###*p* < 0.001 vs TAC group (analyzed using one-way ANOVA).

To determine whether rhein can reduce TAC-induced cardiac hypertrophy in mice, we used echocardiography to measure cardiac function *in vivo*. Compared with the sham operation group, peak flow velocity at the aortic ligation significantly increased, as did diastolic interventricular septal thickness (IVSd), diastolic left ventricular posterior wall thickness (LVPWd), and left ventricular mass (LV Mass), consistent with cardiac hypertrophy having been induced by TAC (Figure 4A; Table 1). In contrast, ejection fraction (EF) and shortening fraction (FS) decreased markedly, and cardiac function was decompensated. Compared with the TAC operation group, EF and FS in the rhein treatment group increased significantly, suggesting that rhein can alleviate the decline in cardiac function caused by TAC. It is worth noting that among these quantities, IVSd most directly reflects the degree of cardiac hypertrophy. Echocardiography showed that compared with the TAC operation group, both the high concentration rhein treatment group and telmisartan positive control group significantly reduced IVSTd. In addition, rhein at 150 mg/kg significantly reduced LVPWd. Although there was no significant difference between low and high doses of rhein in reducing LVPWd, there appeared to be a significant trend toward reduction. Moreover, systolic interventricular septal thickness (IVSs) and systolic left posterior wall thickness (LVPWs) in the rhein group remained at the same level as in the sham group.

**TABLE 1:** Echocardiographic paraments.

Measurements	Sham	TAC	TAC + rhein	TAC + rhein	TAC + rhein	TAC + Tel
			(50 mg/kg)	(150 mg/lcs.)	(200 mecg)	(le mg/kg)
VSd (mm)	0.9110.13	1.2210.23*	1.03 ± 0.20	0.95 ± 0.14	0.9210.10^#^	0.9010.05^#^
LVIDd (mm)	3.5510.19	3.664–0.28	3.3710.23	33,910.44	3.291,032	3.2210.16
LVPWd (mm)	0.8310.08	1.1610.22**	0.9610.14	0.8010.12^##^	0.9810.17	0.840.08^# #^
TVSs (nan)	1.44 ± 0.17	1.610.30	1.55 ± 0.35	1.420.11	1.370.09	1.43 ± 0.14
LVIDs (mm)	2.3610.14	2.350.37	2.3710.47	2.300.46	2.3010.24	1.990.23
LVPWs (mm)	1.08 ± 0.11	1.52 ± 0.17*	1.20 ± 0.19^##^	1.2310.10^#^	1.3510.16	1.21 ± 0.13^#^
LV1asstmg)	90.20 ± 9.52	124.70 ± 2,524*	110.10 ± 30.46	91.81 ± 13.31	96.90112.49	90.99 ± 12.61^#^
EF%	76.6812.13	53.1715.37***	64.33 ± 10 45^#^	69.6216.46^###^	68.1415.07^##^	70.212.20^###^
FS%	44.71 ± 1.99	26.8513 20***	35.1217.46^#^	38.4615.07^###^	37.3213 95^##^	38.7011.70^###^
Heart rate	467.7125.82	479.5146.65	445.5140.61	466.3128.50	444.7142.98	456.2136.85

Data are presented as mean ± SD. *p < 0.05, **p < 0.01, ***p < 0.001 vs sham group, #*p* < 0.05, ##*p* < 0.01, ###*p* < 0.001 vs TAC group. Abbreviations: IVSd: interventricular septum in diastole, LVIDd: left ventricular diastolic internal diameter, LVPWd: left ventricular diastolic posterior wall thickness, IVSs: interventricular septum in systole, LVIDs: left ventricular systolic internal diameter, LVPWs: left ventricular systolic posterior wall thickness, LV mass/BW: left ventricular mass/body weight, EF: ejection fraction, FS: fractional shortening.

Subsequently, anatomical parameters were measured in a mouse model of cardiac hypertrophy induced by the TAC operation. First, we analyzed the ratios of heart weight (HW) to body weight (BW) and tibial length (TL) as indicators of cardiac hypertrophy. As shown in Figures 4C, D, HW/BW (mg/g) and HW/TL ratios (mg/mm) were significantly reduced in the rhein treatment group compared with those in the TAC operation group. In addition, we assessed macroscopic differences in the hearts in response to rhein treatment 28 days after the TAC operation. As shown in Figure 4B, heart size increased significantly in response to the TAC operation, while that of the rhein group and the positive control telmisartan group decreased significantly. Hematoxylin and eosin (H&E) staining and wheat germ agglutinin staining of thin tissue sections further demonstrated that rhein had an inhibitory effect on cardiac hypertrophy induced by the TAC operation in C57BL/6 mice (Figures 4E,F). Next, we used Image-Pro Plus to quantitatively analyze the size (projected area) of cardiomyocytes in the TAC operation and the rhein treatment groups. It was observed that the relative size of cardiomyocytes in the rhein treatment group decreased significantly in a dose-dependent manner (Figure 4H).

**FIGURE 5:**
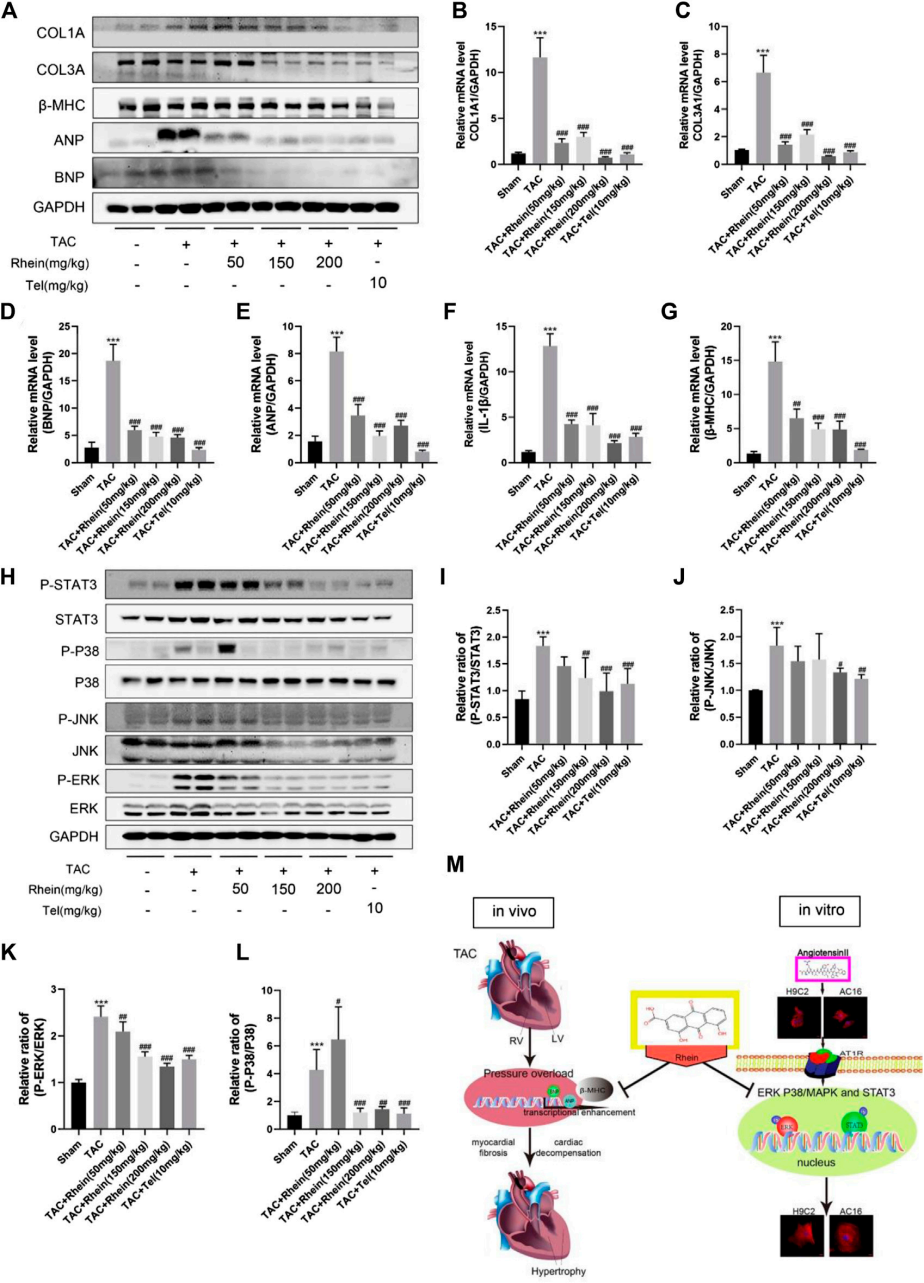
Rhein inhibits TAC operation-stimulated activation of STAT3 and p38/MAPK signaling pathways *in vivo*. **(A)** Relative expression of ANP, BNP, β-MHC, COL1A, and COL3A proteins in the left ventricle treated with TAC operation and rhein, and in the telmisartan positive control group. **(B–G)** mRNA expression of COL1A1, COL3A, ANP, BNP, β-MHC, and IL-1β was examined by RT-PCR. **(H)** The expression levels of signaling pathway proteins (total and phosphorylation of STAT3, JNK, ERK, and p38) were determined by Western blot. **(I–L)** Quantification of the relative changes in the phosphorylation of STAT3, p38, JNK, and ERK. **(M)** Schematic diagram with roles of rhein in TAC operation- and AngII-induced cardiac hypertrophy. Data are mean ± SD (*n* = 6). *p < 0.05, **p < 0.01, ***p < 0.001 vs sham group, #*p* < 0.05, ##*p* < 0.01, ###*p* < 0.001 vs TAC group.

Pathological myocardial hypertrophy is an important risk factor for the development of heart failure and can cause cardiac fibrosis, leading to abnormal cardiac electrical conduction and diastolic dysfunction. To evaluate the effect of rhein on cardiac fibrosis, we stained paraffin plaques with Masson’s trichrome, which stains collagen fibers blue and heart muscle red. As can be seen from stained tissue sections (Figure 4G) and quantitative statistical analysis of areas containing collage fibers (Figure 4I), significant myocardial fibrosis was observed in mice 28 days after the TAC operation. However, the degree of myocardial fibrosis in rhein-treated mice declined in a dose-dependent manner. In addition, we examined the expression of fibrosis marker genes and proteins (Figures 5A–C). Protein levels of the cardiac fibrosis markers, collagen 1A1 (COL1A) and collagen 3A1 (COL3A), increased significantly in the TAC operation group, while rhein inhibited the expression of COL1A and COL3A proteins. Similarly, RT-PCR also showed that COL1A and COL3A mRNA levels were significantly downregulated in the rhein treatment group. Overall, cardiac fibrosis increased in mice 28 days after the TAC operation, and rhein treatment significantly reduced expression of collagen fibers at the mRNA and protein levels.

Based on these experimental results, we propose that rhein has a protective effect on myocardial hypertrophy and cardiac fibrosis induced by a TAC operation *in vivo*. Interestingly, telmisartan, a first-line treatment for hypertension and cardiac hypertrophy, showed similar effects to rhein in improving cardiac function and left ventricular structure.

### Rhein inhibits TAC operation-stimulated activation of STAT3 and p38/MAPK signaling pathways *in vivo*

To investigate the mechanism of rhein on cardiac hypertrophy induced by TAC operation in mice, we examined the effects of rhein on STAT3 and P38/MAPK signaling pathways involved in proliferation and inflammation of cardiac hypertrophy cells. As expected, we found that *in vivo* induced cardiac hypertrophy had the same effect as H9C2 cells in signaling pathway proteins. First, the hypertrophy markers ANP, BNP, and β-MHC were significantly downregulated in the rhein treatment group compared with the TAC Operation group (Figure 5A). The result was further verified by the mRNA level (Figures 5D,E,G). Interestingly, the mRNA level of the inflammatory factor IL-1β was also significantly downregulated in the rhein-treated group (Figure 5F), suggesting that rhein plays a corresponding role in the production of anti-cellular inflammatory factors. In addition, consistent with *in vitro* results, phosphorylation levels of STAT3, JNK, ERK, and P38 were significantly reduced by TAC operation for 28 days after *in vivo* treatment with rhein (Figure 5H). Meanwhile, statistical results showed that the ratios of P-STAT3/STAT3, P-JNK/JNK, P-ERK/ERK, and P-P38/P38 were significantly reduced after rhein treatment with different concentrations *in vivo* (Figures 5I–L). These results suggested that rhein can inhibit the activation of STAT3 and P38/MAPK signaling pathways induced by TAC operation by inhibiting phosphorylation of key proteins.

### Serum biochemical indices showed that rhein had no obvious toxicity and side effects

**FIGURE 6:**
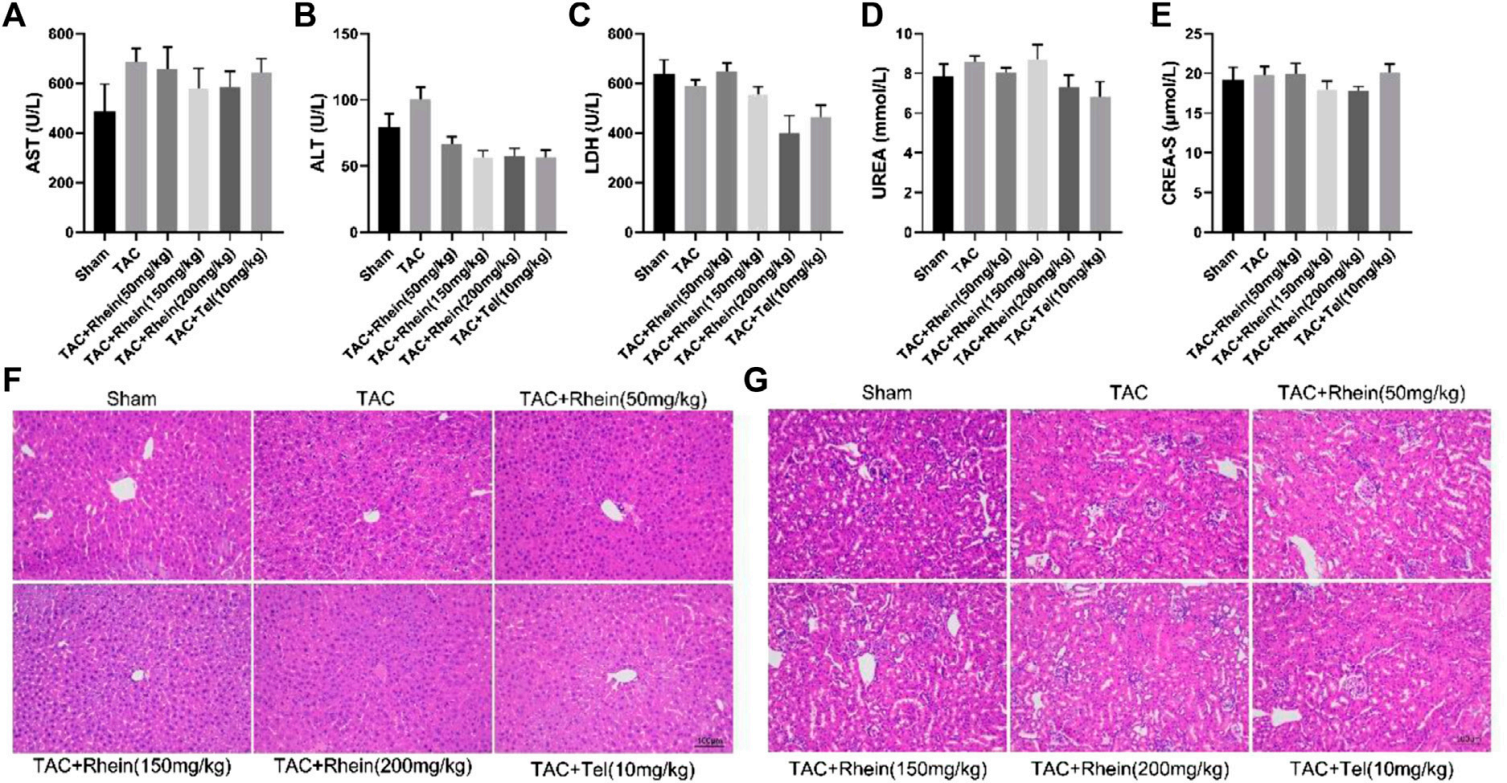
Rhein has no obvious toxicity or side effects on normal organs. **(A–E)** The levels of LDH, AST, ALT, CREA-S, and UREA were tested from the serum of various mice groups. **(F–G)** Liver and kidney histopathology was done by H&E staining. Scale bar = 100 µm. Data are expressed as mean ± SD (*n* = 6). *p < 0.05 vs sham group, #*p* < 0.05, ##*p* < 0.01 vs TAC group (analyzed using one-way ANOVA).

Hepatotoxicity, nephrotoxicity, electrolyte disturbance, and other common drug unexpected adverse reactions were the main hidden dangers of clinical application. Therefore, we tested rhein for possible toxicity or adverse reactions. Serum biochemical indexes such as LDH, AST, ALT, CREA-S, and UREA were determined. As shown in Figures 6A–E, these biochemical indexes in the TAC Operation 28 group increased relatively, but there was no statistical significance. Compared with the sham group, these indexes showed no significant difference after rhein treatment, and even reduced the serum ALT level in mice. These results showed no damage to the liver, kidneys, or heart. In addition, H&E staining analysis supported this conclusion, with no significant hepatic or renal edema, fibrous deposition, hyaline change, and necrosis (Figures 5F,G). Given these findings, we believe that rhein acid had no significant toxic side effects on normal organs.

## Discussion

In our study, we report that rhein induces cardiac hypertrophy *in vivo* and *in vitro* for the first time, which can simultaneously exert anti-hypertrophy effects on rat, mouse, and human cardiomyocytes. In addition, our results proved that rhein can significantly attenuate cardiac fibrosis and inflammation due to cardiac hypertrophy. Our mechanism data showed that STAT3 and P38/MAPK signaling pathways were closely related to the cardiac protective effect of rhein. Rhein not only inhibits the phosphorylation of ERK but also reduces the nuclear enrichment of P-ERK, thus inhibiting the transcription of hypertrophy-related proteins in cardiomyocytes. According to Aoki et al. (Brancaccio et al., 2006; Vallentin and Mochly-Rosen, 2007; Ruppert and Meyer, 2007; O'Rourke et al., 1994), activation of MAPK/ERK and STAT3 can induce increased expression of atrial natriuretic peptide mRNA and protein, resulting in symptoms of cardiac hypertrophy. In this study, specific inhibitors of STAT3 and ERK were used to compare with rhein. It was observed that rhein could inhibit the phosphorylation of STAT3 and ERK as well as the expression of cardiac hypertrophic proteins ANP, BNP, and β-MHC, which had the same effect as the two inhibitors. It was speculated that rhein could target or mainly act on STAT3 and P38/MAPK signaling pathways. Therefore, it played a role in inhibiting cardiac hypertrophy. Moreover, the outstanding findings of this study demonstrated that rhein had no significant toxic and side effects on normal organs. Taken together, our study proved that rhein may be a promising drug candidate for the treatment of cardiac hypertrophy and heart failure.

Myocardial hypertrophy is an important risk factor for the progression of heart failure, which is often accompanied by fetal gene activation, electrophysiology, and diastolic dysfunction, and ultimately leads to heart injury and cardiac insufficiency. At present, although there are many reports on the pathophysiological mechanism of cardiac hypertrophy, the clinical treatment effect for patients with cardiac hypertrophy is limited. Therefore, it is necessary to find other new effective drugs to prevent and treat cardiac hypertrophy. Rhein is a monomer compound found in many medicinal plants such as *rhubarb* and *Polygonum multiflorum* (Legendre et al., 2007; Ma et al., 2021). There is growing evidence that rhein has a positive therapeutic effect on different disease models. For example, rhein can resist a variety of malignant tumors and promote tumor cell apoptosis (Cho et al., 2017; Henamayee et al., 2020). Guo and Liu et al. found that rhein can inhibit renal tubular epithelial hypertrophy and extracellular matrix accumulation (Guo et al., 2001). Gao et al. also found that rhein has a protective effect on diabetic nephropathy and it also improved dyslipidemia (Gao et al., 2010). In addition, studies by Chen and David et al. showed that rhein attenuates fibrosis in the kidney, myocardium, and liver through multiple pathways (Guo et al., 2002; Chen et al., 2019; Barbosa et al., 2020). Other studies have shown that rhein can inhibit influenza virus transmission by inhibiting TLR4, MAPK, and NF-KB signaling pathways (Wang et al., 2018). At present, it is not known whether rhein has a therapeutic effect on cardiac hypertrophy and cardiac insufficiency. In our experiment, we investigated the relationship between rhein and cardiac hypertrophy based on cellular and animal models. Telmisartan is traditionally one of the AngII receptor blockers (ARBs) that has been clinically used for the treatment of hypertension. Hypertension is known to increase ventricular afterload, which promotes cardiac hypertrophy. Li et al. showed that telmisartan could downregulate ANP and BNP expression by inhibiting the NFAT signaling pathway (Li et al., 2017), which effectively inhibits cardiac hypertrophy. Therefore, telmisartan is used as a positive control to evaluate the therapeutic effect of rhein in this model of cardiac hypertrophy. We found that rhein inhibited AngII- and TAC operation-induced cardiac hypertrophy *in vitro* and *in vivo*, respectively. The therapeutic value of large doses of rhein is comparable to that of telmisartan.

Cardiac hypertrophy accompanied by accumulation of extracellular matrix collagen fibers seriously affects the normal diastolic function of the heart, resulting in decreased ejection fraction and heart failure (Molkentin et al., 1998; Burchfield et al., 2013). According to previous studies, collagen fibers COL1A1 and COL3A1 play an important role in cardiac hypertrophy. Yao et al. found that inhibition of fibrosis can significantly improve cardiac hypertrophy (Yao et al., 2020) in which collagen fibers 1 and 3 are significantly increased after TAC operation and return to normal level after inhibiting the fibrosis pathway. In our study, rhein had a significant effect on the improvement of fibrosis after TAC operation, and the area of fibrosis decreases in a dose-dependent manner. In general, rhein also played an anti-fibrosis role in the treatment of cardiac hypertrophy.

A TAC operation leads to cardiac hypertrophy through ventricular wall pressure overload, while AngII promotes cardiac hypertrophy through vasoconstriction, activation of the RAS, water and sodium retention, and cardiotoxicity, all of which ultimately activate the STAT3 and p-38/MAPK signaling pathways (Pan et al., 1997; Brancaccio et al., 2006). According to previous research, rhein can sensitize human pancreatic cancer cells to EGFR inhibitors by inhibiting the STAT3 pathway (Yang et al., 2019). In addition, Hao et al. found that rhein improved motor function in rats with spinal cord injury by inhibiting the P38/MAPK pathway (Hao et al., 2019). Ma et al. showed that rhein can affect the malignant phenotype of renal cancer by inhibiting MAPK/NF-κB signaling (Ma et al., 2018). In the present study, we clearly demonstrated that rhein can significantly reduce heart muscle hypertrophy *in vivo* and *in vitro* and inhibit the expression of P-STAT3, P-P38, P-JNK, and P-ERK. More importantly, rhein inhibited phosphorylation and nuclear enrichment of ERK and STAT3. Based on these results, we conclude that rhein can be targeted to alleviate TAC operation- and AngII-induced cardiac hypertrophy by inhibiting the STAT3 and P38/MAPK signaling pathways. However, the molecular sites where rhein targets ERK or STAT3 remain to be studied.

There have also been many reports on the protective effect of rhein on other tissues and organs. Hu et al. found that rhein can protect renal function and improve the median survival time of SAMP10 mice (Hu et al., 2013). In addition, Liu et al. confirmed that rhein can protect pancreatic B cells and inhibit apoptosis under hyperglycemic conditions (Liu et al., 2013). In our experiment, we also explored the effects of rhein on various organ functions, and the results showed that rhein did not exert toxic effects on electrolytes and the liver, kidney, and heart at therapeutic doses.

In conclusion, this study has shown for the first time that rhein can significantly inhibit myocardial hypertrophy induced by a TAC operation in mice and AngII exposure in cardiomyocytes. In addition, rhein can protect the heart by reducing cardiac fibrosis, inhibiting the activation of STAT3 and P 38/MAPK signaling, and phosphorylation and nuclear enrichment of ERK, thereby reducing transcription of hypertrophic proteins. Finally, we demonstrated that rhein had no significant toxicity or side effects in normal cells and organs. Therefore, the natural product rhein may become a new effective drug in the treatment for cardiac hypertrophy and heart failure.

## Data Availability

The raw data supporting the conclusion of this article will be made available by the authors without undue reservation.
